# Assessing Changes in the Ecosystem Services Value in Response to Land-Use/Land-Cover Dynamics in Shanghai from 2000 to 2020

**DOI:** 10.3390/ijerph191912080

**Published:** 2022-09-24

**Authors:** Yuan Gong, Mengmeng Cai, Lei Yao, Linsong Cheng, Chunxu Hao, Zheng Zhao

**Affiliations:** 1 School of Tourism, Shanghai Normal University, Shanghai 200234, China; 2 Shanghai Institute of Tourism, Shanghai 201418, China; 3 School of Economics and Management, Beijing Forestry University, Beijing 100083, China; 4 Chinese Academy for Environmental Planning, Beijing 100012, China

**Keywords:** coefficient of sensitivity, ecosystem services value, land-use/land-cover dynamics

## Abstract

Land resources are foundational for human survival and development. In contrast, land use/land cover (LULC) dynamics drive considerable changes in ecosystem services. Recently, China witnessed a new stage of rapid urbanization. Therefore, investigating the relationships between ecosystem services value (ESV) and LULC in these areas is highly relevant. Based on the data of land use and socioeconomic development in Shanghai from 2000 to 2020, we adopted a land use/land cover dynamics analysis method and established the ESV per unit area at the city scale, discussed the impact of LULC on ESV spatially and quantitatively, and tested the research process based on the sensitivity analysis of the ESV coefficient. The results show that from 2000 to 2020, the LULC pattern in Shanghai rapidly changed. In particular, the area of cultivated land has shrunk by 123.96 thousand hm^2^, while the construction land has expanded by 141.26 thousand hm^2^, which has led to a decline in ESV of the entire city (especially regarding hydrological adjustment and biodiversity). Nevertheless, although the area of trench and lakes only occupies 1.67–3.16% of the total area of land, its ecological value accounts for an astonishing 23.80–50.70% of the total ESV. At the district level, the primary decline in eco-system services value was noted in the Chongming District in the north and Pudong New Area in the east of Shanghai. However, due to the overall planning of the city and the advantages of its resource endowment, Qingpu District and its surrounding areas in western Shanghai have witnessed improvements in terms of the values of hydrological adjustment, water supply, and environmental purification. This study presents a deeper and more comprehensive understanding of issues regarding ESV in rapidly urbanized areas, thereby providing an important reference for decision-makers regarding the rational layout of cities, sustainable use of land, and management of natural ecosystems.

## 1. Introduction

The ecosystem is composed of plants, animals, microbial communities, and the abiotic environment. The ecosystem itself and the services provided by its species are the conditions and processes needed to meet and sustain human production and life. These provide a wide range of direct and indirect services for human survival and contribute significantly to improving human well-being [1,2,3,4,5]. Since the 1990s, many studies have been conducted to explore ecosystem service values, including in areas such as biological resources, biodiversity conversion, and the management of tropical forests, nature reserves, and endangered species [6,7,8,9,10,11,12]. Despite the incredible contribution of ecosystem services to natural functions in addition to mankind’s sustainable well-being and survival, these have been significantly degraded over time and space globally [13,14,15,16,17,18,19]. Over the past 50 years, human beings have caused changes in ecosystem services much faster and more widely than in any other period in human history [20]. One of the most important impacts of human activities on variable ecosystems is the change in land use, which not only alters the layout of vegetation and landscape but also the structure and function of ecosystems, thereby impacting ecosystem services value (ESV) [21]. The change in land-use and land-cover (LULC) has featured in current research related to global environmental change and has been widely recognized as the main driving force for significantly changing ecosystem services [22,23,24,25,26,27,28,29]. LULC widely impacts ecosystem services. As such, its abnormal changes may bring about serious consequences, such as ecosystem integrity reduction and habitat and biodiversity loss, thus reducing the level of ecosystem services [30,31,32,33]. Accordingly, the degradation of ecosystem services also affects the structure and efficiency of land use, forming a vicious cycle of ecological-economic systems [34,35,36]. Therefore, measuring the changes in ecosystem services caused by land-use dynamics has become an effective tool for assessing the environmental costs and benefits of related policies and plans.

From an economic perspective, measuring the impact of LULC on various ecosystems is an effective way to assess the environmental costs and benefits of different land-use plans and decisions. Based on the evaluation model of ecosystem services value established by Costanza et al., scholars from home and abroad have conducted extensive empirical studies [37,38,39]. Millennium ecosystem assessments show that ESV is a critical indicator that reflects regional sustainable development and indicates the significance of its inclusion in decision-making [40]. It has been indicated that the analysis of change in ESV is an important tool for helping make decisions related to sustainable land use [41,42]. These studies mainly focus on the impact of LULC on ESV, and are greatly expanded at different spatial scales, as well as evaluation models and methods [43,44,45,46]. However, these studies also have inherent limitations, which have been comprehensively analysed and discussed in the work of [47,48,49].

Compared with other parts of the world, LULC dynamics in China are substantially more complicated. With the rapid development of China’s economy and its increasing urbanization, land resources in China have undergone huge conversions and occupations, resulting in a drastic transformation of land-use types and areas, which have significantly impacted the value of regional ecosystem services [50,51,52,53]. China’s expanding urban area has caused the loss of a large amount of cultivated land [54]. Chinese scholars have paid more attention to the estimation of ESVs [55,56,57,58]. Xie et al. [59,60,61] further improved the equivalent factor system of China’s ESV, achieving the goal of comprehensively evaluating China’s ecosystem and its services value. This system has been widely adopted and used. Many of these studies have evaluated the ESV of forests, wetlands, and water in urban areas [62]. In this process, a research method based on the landscape layout or LULC has become important for measuring the impact of urban expansion on ecosystem services [63,64]. At present and in the future, we should study the influence of LULC on ESV, consider how to further facilitate the benign interaction between urban development and the ecosystem, and accelerate green development and transformation. All of these must be emphasized amid urbanization since it is significantly important to adjust and optimize land use patterns and then promote and coordinate regional sustainable development [65,66].

There are many studies have been conducted regarding LULC in China, including its dynamics in urbanized areas [67,68,69,70,71,72,73]. In recent years, China’s deepening urbanization, the mounting pressure of its large population, and the correspondingly soaring need for urban land caused by increasing human activities have led to huge LULC dynamics. Monitoring LULC dynamics and its impact on ESV are crucial for decision-making regarding the rational layout of cities, the sustainable use of land, and the management of natural ecosystems in China. Therefore, given the urgent need for such quantitative analysis in Chinese cities, we aim to estimate the impact of LULC on ESV in Shanghai from 2000 to 2020 by using a 30-m fine-resolution land cover dataset to narrow the gap regarding LULC dynamics in existing studies.

In the 20 years from 2000 to 2020, Shanghai’s economy grew more than 15 times. Simultaneously, Shanghai’s rapid development has also been accompanied by resource consumption, environmental deterioration, and transformation of land use patterns. From this perspective, land use/land cover in Shanghai has significantly changed, both temporally and spatially, which needs special attention. In recent years, documents emphasizing the optimization of the systematic structure and balanced development of the city, strengthening urban land use, and promoting the coordinated development of industrial layout, spatial structure, and ecological environment have been issued, such as the Shanghai City Master Plan (2017–2035), Shanghai Land Use Master Plan (2010–2030), and other documents have been issued. In this study, we derived the multi-period LULC monitoring dataset of China from Landsat MSS, TM/ETM, and Landsat 8 satellite remote sensing data and combined it with relevant statistical data. Based on this, we selected the representative city of Shanghai to study LULC and its influence on ESV. The degree of land-use dynamics, ecosystem services value, contribution rate, coefficient of sensitivity, and other indicators were used to objectively reflect the influence of LULC on ESV in Shanghai from 2000 to 2020. The purpose of this study is to provide a deeper and more comprehensive understanding of the issues related to ESV in rapidly urbanized coastal areas, thus providing theoretical and methodological support for promoting the sustainable development of land resources and social economy in Shanghai. Specifically, our study aims to answer three main questions: (1) How did the LULC dynamics occur in Shanghai from 2000 to 2020? In this regard, we will analyse and discuss the specific changes of the LULC dynamics in Shanghai; (2) How did the total and individual ESV change in response to the LULC dynamics during this period? This is the key issue of this study, and we will try to explore and reveal the impact relationship between them; (3) Regarding LULC dynamics, what management strategies should Shanghai adopt? In summary, this study aims to address these issues and assumes that LULC dynamics play a key role in the degradation of natural ecosystem services over time.

## 2. Materials and Methods

### 2.1. Study Area

**Figure 1:**
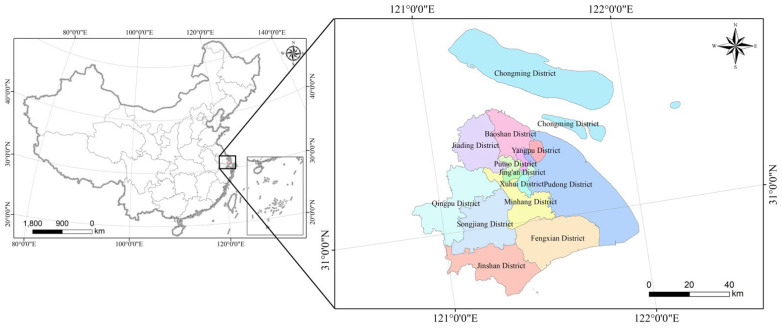
Map showing the location of Shanghai, its geopolitical zones, and major districts.

As shown in Figure 1, Shanghai, located in eastern China (between 120°52′–122°12′ E and 30°40′–31°53′ N), is part of the alluvial plain of the Yangtze River Delta, with an average height of 2.19 m above sea level and an area of 6340 km^2^. Situated in a humid subtropical region, Shanghai has a typical subtropical monsoon climate. It features four distinct seasons, and is rainy and hot during the same period (i.e., the average temperature of the coldest month in winter is above 0 °C, and it is hot and rainy in the summer). As the economic, financial, and commercial centre of China, Shanghai is a region that boasts the strongest comprehensive and economic strengths in China. In 2020, the GDP of Shanghai was 3870.058 billion yuan, ranking first among the cities in China (excluding Hong Kong, Macao, and Taiwan). In recent decades, Shanghai has undergone intensive land use dynamics owing to rapid urbanization, which has aroused public and academic attention on the need to study changes in ESV caused by LULC in Shanghai. 

### 2.2. Data Sources

Based on the National Resources and Environment Database and Landsat remote sensing image data of the United States as the main information source, the Chinese Academy of Sciences (CAS) established the national 1:10 scale multi-period LULC dataset, namely the China Land Use and Cover Change (CNLUCC) through artificial visual interpretation, and adopted a conical projection of Albers positive axis equi-area double standard parallels as the data projection system. In this study, the data used to analyze the impact of LULC on ESV in Shanghai are based on five land-use maps generated by the Institute of Geographic Sciences and Natural Resources Research, CAS, which are from 2000, 2005, 2010, 2015, and 2020. In this study, Landsat Thematic Mapper (TM), Enhanced Thematic Mapper Plus (ETM+) remote sensing image data were mainly used for remote sensing interpretation of data from 2000, 2005, and 2010, while Landsat 8 remote sensing image data were used for updating the LULC data from 2015 and 2020.

Based on the national standard stipulated in China’s Land Use Classification in 2017 (GB/T 21010-2017), land use types in Shanghai, after comprehensive consideration of the characteristics of its land resources, were classified as cultivated land, forest land, grassland, trench and lakes, wetland pits, and construction land in ArcGIS10.2, which can highlight the characteristic land-use types of coastal areas (Table 1). Based on the relevant calculation rules of ecosystem services value, construction land with ecosystem services value equivalent to less than 0 was excluded in this study. The output value of agricultural commodities of grain crops (100 million yuan), sown area of grain crops (10,000 hm^2^), and the consumer price index (CPI) used in ecosystem services value calculations were obtained from the Shanghai Statistical Yearbook (2001–2021) and the China Statistical Yearbook (2001–2021).

### 2.3. Methods

#### 2.3.1. Land Use/land Cover Dynamics Analysis

Land use dynamic degree (LUDD) describes the change rate of land use in a certain period. The comprehensive LUDD is often used to measure the change rate of land use over an entire area, whereas the individual LUDD is used to calculate the change rate of a certain land type. The calculation method for LUDD is as follows: the ratio of the total area transformed from one land type to another to the initial area of the land type transformed in a certain period [74]. The larger the index value, the greater the land-use change, and vice versa.

(1) $$M_{mn}=\left(LU_{ib}-LU_{ia}\right)/\left(LU_{ib^{\prime }}-LU_{ia^{\prime }}\right),\;LUDD_{i}=\left(\left(LU_{ib}-LU_{ia}\right)/LU_{ia}\right)/T$$

In the formula above, *LU_ib_* is the final value of land type *i* in period *m*, *LU_ia_* is the initial value of land type *i* in period *m*, while *LU_ib’_* is the final value of land type *i* in period *n*, *LU_ia’_* is the initial value of land type *i* in period *n*, and *M_mn_* is the ratio of the change in period m to that of period *n*. *LUDD_i_* is the dynamic degree, and T is the study period, the unit of which is set as year in this study; hence, the *LUDD_i_* in the formula represents the average annual change rate of a certain land type, and the comprehensive LUDD equals the sum of *LUDD_i_*.

#### 2.3.2. Assignment of Ecosystem Service Values

In this study, the output value of agricultural commodities of food crops (100 million yuan) and the sown area of food crops (10,000 hm^2^) in Shanghai from 2000 to 2020 were used to estimate the economic value of food crops (yuan hm^−2^) in Shanghai. The ecosystem services value equivalent factor is used to represent the relative contribution rate of the potential ecosystem services value. Without considering human input, the economic value provided by natural ecosystems is approximately 1/7 of the economic value produced by existing food crops per unit area [75]. Therefore, the single equivalent ESV of the Shanghai ecosystem (yuan·hm^−2^) was modified. To make the raw data comparable, the CPI was used to adjust the ecosystem services value of different years to the level based on data from the year 2000 (Table 2). The calculation results show that the average value of a single equivalent ecosystem service in Shanghai from 2000 to 2020 was 1932.63 yuan·hm^−2^.

Based on the ecosystem services value coefficient determined by Costanza et al. and combined with the equivalent conversion method of Chinese ecosystem value proposed by Xie et al. [59,60,61], this study determined the ESV per unit area of land ecosystem in Shanghai after appropriate adjustment to connect various land types with the closest ecosystem types. Therefore, ecosystem services in Shanghai can be divided into four categories and 11 subcategories: supply services, regulating services, support services, and cultural services. The ecosystem service value equivalent of each land use type was selected according to the following principles: (1) cultivated land, corresponding to farmland, equals the average value equivalent of dry land and paddy fields; (2) forestland, corresponding to forest, equals the average value equivalent of the four ecosystem systems: conifer, conifer and broadleaf mixed, broadleaf, and shrub; (3) grassland corresponds to irrigated grass; trench and lakes (including rivers, ditches, and lakes) correspond to water systems; (4) wetland pits (pits, mudflats, and beaches) correspond to wetlands. In addition, the ESV of urban and rural land, industrial and mining land, and residential land are excluded [61]. Therefore, the coefficients of ESV (yuan·hm^−2^) of each land use type in Shanghai can be obtained as shown in Table 3.

#### 2.3.3. Calculation of the Ecosystem Service Value

Based on existing studies, in the formula for calculating ecosystem services value, ESV represents the total value of the regional ecosystem services. *A_i_* refers to the area of land use type *i*, and *V_ij_* represents the value per unit area produced by ecological services j of land use type *i*. m refers to the sum of all land use types, and n represents the sum of all ecosystem service categories.

(2) $$\mathrm{ESV}=\sum_{i=1}^{m}\sum_{j=1}^{n}A_{i}V_{ij}$$

To facilitate the analysis of changes in ecosystem services value and the comparison of ESV in different regions, this study, based on the research of traditional calculation methods of ESV conducted by Zhang et al. [76,77], introduces the contribution rate (CR) of ecosystem services value, which is used to describe the impact of the change in the value of individual ecosystem services on the total value of ecosystem services. *CR_i_* is the ratio of the value of ecosystem service *i* to the total value of ecosystem services, whereas *CR_j_* equals the ratio of the value of ecosystem type *j* to the total value of ecosystem services.

(3) $$CR_{i}=ESV_{i}/ESV,\;CR_{j}=ESV_{j}/ESV$$

#### 2.3.4. Sensitivity (CS)

The coefficient of sensitivity is a type of elastic coefficient, which is used to show the dependence of ecosystem service value on the ESV coefficient. That is, the degree of change in ESV is caused by a 1.0% change in the ESV coefficient [78,79]. In this study, the value coefficients of various land-use types in Shanghai were adjusted by ±50.0% (the results are the same) [80,81]. The formula for calculating the coefficient of sensitivity is as follows:

(4) $$\mathrm{CS}=\left(\left(ESV_{2}-ESV_{1}\right)/ESV_{1}\right)/\left(\left(V_{2i}-V_{1i}\right)/V_{1i}\right)$$

where CS is the coefficient of sensitivity of each ESV coefficient in the study area. *V*_1*i*_ and *V*_2*i*_ represent the ESV per unit area produced by land use type *i* before and after adjustment, respectively, while *ESV*_1_ and *ESV*_2_ represent the total ESV before and after adjustment, respectively. If the ESV is inelastic relative to V, then CS < 1, meaning that the ESV is insensitive to the ESV coefficient. Conversely, if ESV is elastic relative to V, then CS > 1, indicating that ESV is sensitive to the ESV coefficient. As CS increases, its ESV becomes more dependent on the ESV coefficient, indicating that the accuracy of the ESV coefficient matters.

## 3. Results

### 3.1. Land-Use/Land-Cover Patterns in Shanghai

**Figure 2:**
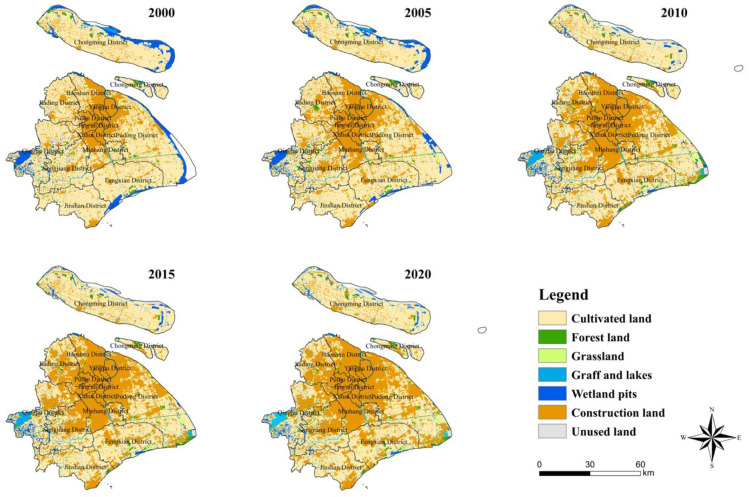
Spatial Distribution of LULC in Shanghai from 2000 to 2020.

From 2000 to 2020, the main types of land use in Shanghai were cultivated land, trench, lakes, and construction land, and the sum of the three areas in the five years accounted for over 90% of the total area of Shanghai, as shown in Figure 2.

From 2000 to 2020, Shanghai witnessed rapidly changing LULC patterns and a significant decline in the area of cultivated land, trench, and lakes, with a net decrease of 123,960 hm^2^ of cultivated land. Meanwhile, the net increase in construction land ranked first, reaching 141,260 hm^2^. The increase in construction land is approximately equal to the decrease in cultivated land, forest land, trench, and lakes, as shown in Table 4. During this period, in addition to rapid urbanization and industrialization, Shanghai experienced a greater impact of human activities on land use, which is shown by the fact that more construction land caused by urban expansion has encroached on cultivated land, forest land, and trench and lakes; thus, influencing ESV. Conversely, the degree of LULC dynamics reflects the speed of land use change over a certain period and indicates the impact of human activities on the natural environment. The calculated results demonstrate a substantial change in LUDD for different land-use types in Shanghai from 2000 to 2020. In particular, the LUDDs of construction land, trench and lakes, forest land and grassland were positive, indicating that the areas of these land types increased significantly. In contrast, the LUDDs of cultivated land and wetland pits were negative, indicating that the area of these three decreased.

In the Shanghai Main Functional Area Planning issued by the Shanghai government, the development strategy for the vast area in the west and south of the city is divided into new areas, whose development focus is intensive and efficient, with improved functions, a friendly environment, a harmonious society, and the integration of urban and rural areas. In other words, the increase in lakes in Shanghai is probably due to the promotion of relevant policies. This study will further explain this point.

### 3.2. Estimated Changes in Ecosystem Services

**Figure 3:**
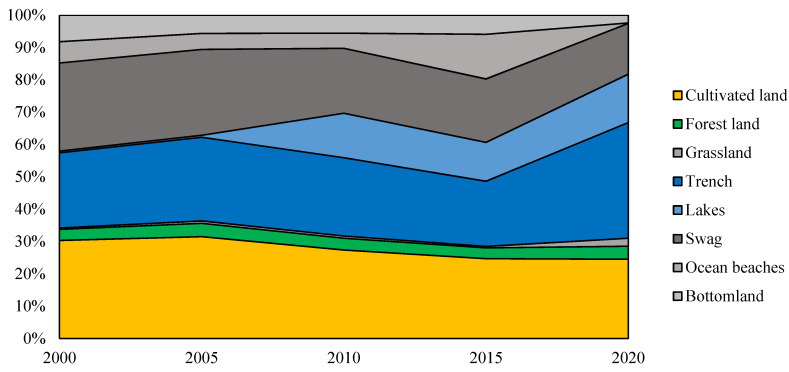
The proportion of ecosystem services provided by different land use types from 2000 to 2020.

Ecosystem services values of Shanghai in 2000, 2005, 2010, 2015, and 2020 were 11,458.78 million yuan, 10,269.72 million yuan, 10,539.58 million yuan, 11,148.40 million yuan, and 10,320.38 million yuan (Table 5), respectively, demonstrating an overall downward trend (−1138.40 million yuan). Specifically, the ESVs of wetland pits and cultivated land saw the biggest decrease of 2933.43 million yuan and 946.29 million yuan, respectively. Simultaneously, the contribution rate of wetland pits decreased by 23.78% during the 20-year period. Although the ESV of trench and lakes, and grassland increased by 2505.79 million yuan and 211.81 million yuan respectively, the increase of its contribution rate was approximately equal to the decrease of that of wetland pits (Figure 3). Nonetheless, it cannot counter the loss of ESV of wetland pits and cultivated land in terms of absolute value, which also reflects the importance of wetland pits in the entire ecosystem. Among all land use types, wetland pits, trench and lakes, and cultivated land generate the most ecological value, accounting for over 90.0% of the total, whereas forest land and grassland produce rather little ecological value. In addition, although the area of trench and lakes in Shanghai only occupies 1.67–3.16% of the total area of land, its ecological value accounts for an astonishing 23.80–50.70% of the total ESV. As such, we can see that the ecological and landscape effects of trench and lakes are very prominent in Shanghai (the eastern coastal area). However, considering the decrease in the contribution rate of wetland pits, the advantage of providing ecosystem service value is gradually being undermined, accompanied by a certain degree of destruction, which puts an invisible strain on the health of Shanghai’s urban ecosystem in the long run.

The change in ESV caused by LULC can also be analyzed from the perspective of conversion between various land use types (Table 6). First, the main reason for the declining ESV trend from 2000 to 2020 was the shift from cultivated land and wetland pits to construction land. During the urban expansion of Shanghai, the extended construction land has greatly encroached on cultivated land and wetland pits, which has indirectly led to a decline in the ESV of the entire city. Based on Table 4, we can see that the area of wetland pits in 2000 accounted for 7.15% of total area of Shanghai, but this figure decreased to 2.75% in 2020. In addition, the ecosystem services value provided by wetland pits accounted for approximately 18.28–42.06% of the total ESV of Shanghai. Due to large-scale urban construction, Shanghai has witnessed significant space exploration in recent years. With the rapid growth of urban construction land and the relentless expansion of urban areas, wetland pits have been shrinking annually, resulting in declining ESV. On this point, previous studies have reached similar conclusions, namely that wetland reclamation reduces the diversity of wetland patch types, leading to the reduction or even loss of wetland biodiversity, which is also the reason for the significant decline in ESV [82,83].

Meanwhile, the shift from wetland pits to trench and lakes contributed to increasing the ESV of the entire city from 2000 to 2020, especially from 2005 to 2010. Similarly, by virtue of the ecological civilization construction strategy advocated by the Chinese government in recent years, the shift from construction land to other land use types greatly facilitated the increase in ESV in 2015–2020. During this period, Shanghai conducted a large number of ecological restoration and environmental protection projects, such as the development of the Chongming ecological island, construction of the Huangpu Riverside greenway. Efforts have also been made to further develop the Shanghai Jiuduansha Wetland National Nature Reserve, Shanghai Chongming Dongtan National Nature Reserve, and other regional projects related to ecological environmental protection. Therefore, great strides have been made regarding ecological restoration and environmental protection in Shanghai in recent years, controlling the scale and mode of urban construction to a certain extent, especially adjusting, and optimizing the structure of urban land use, and controlling wetland pit contraction.

### 3.3. Composition of Ecosystem Service Values

According to Table 7, over the years, the ecosystem service values of Shanghai has presented an average trend of “regulating service > support service > supply service > cultural service”. The ESV of hydrological regulation has accounted for over half of the total service value for many years, which is closely related to the type of ecosystem in the eastern coastal region. Correspondingly, the value of regulating ecosystem services is relatively high, with hydrological regulation, gas regulation, climate regulation, and environmental purification increasing from high to low in terms of the ESV generated. From 2000 to 2020, from the perspective of time, barring the water supply and hydrological adjusting service, has partially improved, the value of all of the ecological services demonstrated a downward trend to varying degrees. In particular, the ESV of biodiversity has been greatly decreasing, exerting a negative impact on the total ESV of Shanghai, which is noteworthy. This can be explained by, such as deepening urbanization, enlarging urban population, and pollution caused by urban development.

For the convenience of analysis, we selected seven districts: Huangpu, Xuhui, Changning, Jing’an, Putuo, Hongkou, and Yangpu as the city center. In general, choosing the seven districts west of the Huangpu River as the core area was based on the geographical characteristics and historical evolution of Shanghai. After the signing of the Treaty of Nanjing between China and Britain in 1842, Shanghai officially opened as one of the five trade ports. Since the 1990s, large-scale renovation of infrastructure has been performed, rendering this region one of the most prosperous areas in Asia, which is also the core area of Shanghai.

**Figure 4:**
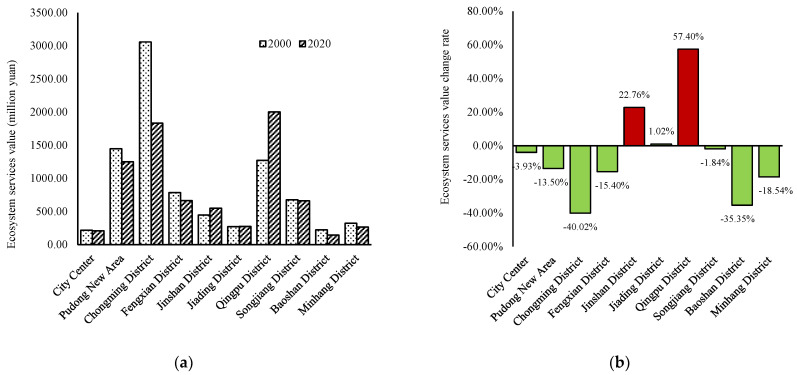
(**a**) Ecosystem services value; (**b**) 2000–2020 ecosystem services value change rate.

The calculation results for the total ESV based on the analysis of each district in Shanghai are shown in Figure 4. We can see that Chongming District exhibited the highest ESV in 2000 (4017.21 million yuan), followed by Pudong New Area (1900.00 million yuan), Qingpu District (1672.88 million yuan), Fengxian District (1031.23 million yuan), Songjiang District (887.32 million yuan), Jinshan District (586.86 million yuan), Minhang District (426.54 million yuan), Jiading District (356.77 million yuan), Baoshan District (294.39 million yuan), and city center (285.56 million yuan). We can see that, barring Chongming District, ESV in 2000 was generally lower in the north of Shanghai, especially in the city center, which was largely since most of the land was used for construction purposes in this area in 2000. From 2000 to 2020, ESV mainly increased in Qingpu District, Jinshan District and Jiading District, but decreased in other regions, with the highest decline rate (−40.02%) in Chongming District, followed by Baoshan District (−35.35%) and Minhang District (−18.54%).

### 3.4. Spatial Patterns of Change in the Value of Ecosystem Service Functions

**Figure 5:**
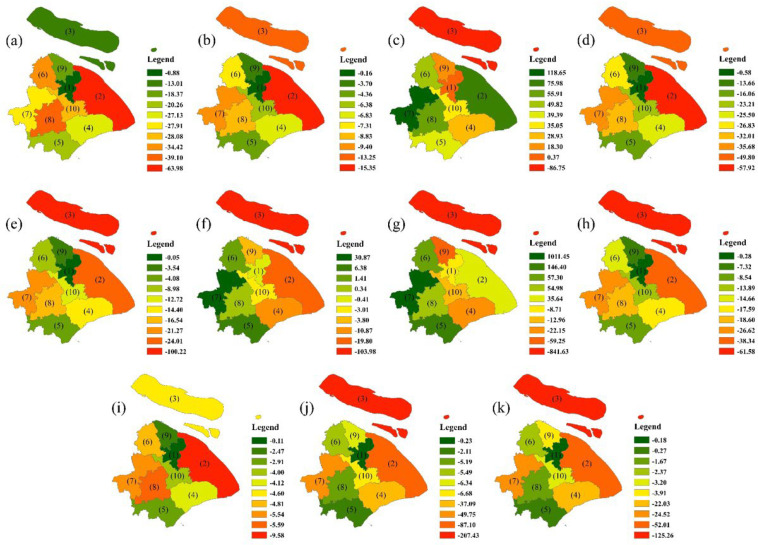
Spatial patterns of value change rate of ecosystem service functions (%): (**a**) food production, (**b**) material production, (**c**) water supply, (**d**) gas regulation, (**e**) climate regulation, (**f**) environment purification, (**g**) hydrological adjusting, (**h**) soil conservation, (**i**) nutrient cycling, (**j**) biodiversity, (**k**) landscape. (1) City Center, (2) Pudong New Area, (3) Chongming District, (4) Fengxian District, (5) Jinshan District, (6) Jiading District, (7) Qingpu District, (8) Songjiang District, (9) Baoshan District, (10) Minhang District.

The rate of change of the ecosystem services value in Shanghai from 2000 to 2020 is shown in Figure 5. Overall, the ESV of all districts in Shanghai showed a downward trend, with Chongming District in the north and Pudong New Area in the east as the main factors. Specifically, the Pudong New Area has witnessed the greatest decrease in food production, material production, gas regulation, and nutrient cycling; while Chongming District has experienced the greatest drop in water supply, climate regulation, environmental purification, hydrological adjustment, soil conservation, biodiversity, and landscape. In particular, the decline of hydrological adjusting (−841.63 million yuan) and biodiversity (−207.43 million yuan) is much higher than that of other regions, indicating that the loss of its ESV was the most serious. Conversely, barring Chongming District, the value of water supply has increased in all other districts, especially Qingpu District (+118.65 million yuan) in the west of the city. The value of hydrological adjustment in Qingpu District has risen significantly (+1011.45 million yuan) and that of environmental purification has also increased (+30.87 million yuan).

It should be noted that, located in the lower reaches of Taihu Lake and the upper reaches of the Huangpu River, Qingpu District boasts 1817 rivers, stretching 2155 km, and 21 lakes, covering a total area of 59.3 km^2^. It is rich in natural resources that can provide high values for hydrological adjustment, water supply, and environmental purification. Qingpu District and its surrounding Jiading District, Jinshan District, Songjiang District, and Fengxian District are all classified as new urbanized areas in the Shanghai Main Functional Area Planning issued by the Shanghai Government. The development strategy for this area is intensive and efficient, with improved functions, a friendly environment, a harmonious society, and the integration of urban and rural areas. Therefore, the increase in the ESV of Qingpu District also benefits from the support of relevant policies.

### 3.5. Coefficient of Sensitivity (CS) Analysis Results

By adjusting the ESV coefficient per unit area of each land use type by ±50.0%, the CSs of ESV in Shanghai in the corresponding years can be obtained through calculation (Table 8). We can see that the coefficients of sensitivity of all land use types in Shanghai from 2000 to 2020 are all less than 1, indicating that the ESV is inelastic to the ESV coefficient (V) per unit area. This shows that the ESV coefficient adopted in this study is in line with the actual situation in Shanghai, so the calculation results are also credible. Specifically, the CS of trench and lakes increased significantly from 2000 to 2020 (+0.269), while the CS of wetland pits decreased significantly (−0.238), indicating that the change in ESV coefficients of trench and lakes can amplify the total ESV, whereas the change in that of wetland pits has the opposite effect. Nonetheless, the CS of cultivated land, forestland and grassland is close to 0, and the change in ESV has little influence on the total ESV coefficient despite its trend of fluctuation.

## 4. Discussion

### 4.1. Impact of LULC Change on Ecosystem Services in Shanghai

Existing studies clearly show that in large cities such as Shanghai, the most evident temporal and spatial change in land use type can be attributed to the expansion of construction land and diminishing natural vegetation [84]. Based on the measurement method and research results on the ESV by Costanza et al. [15], we conclude that the total ESV in Shanghai decreased by 9.93% (−1138.40 million yuan) from 2000 to 2020, resulting from the expanding area of construction land, and the shrinking cultivated land and wetland pits. Some studies have pointed out that wetlands regulate floods, replenish groundwater, improve the climate, purify pollution, and maintain the balance of the regional ecosystem, which is the most important biological landscape and living environment for human beings [85,86]. Specifically, the ESVs of trench and lakes, and grassland have grown by 2505.79 million yuan and 211.81 million yuan, respectively. The increase of their contribution rate is approximately equal to the decrease of that of wetland pits. However, in terms of absolute value, this cannot make up for the loss of ESV of wetland pits and cultivated land. From 2000 to 2020, the decreasing ESV at the level of each district in Shanghai, can be explained by the decrease in ESV of Chongming District in the north and Pudong New Area in the east, which may be owing to the increase in construction land in the northern part of the city. During the study period, Chongming District, located in the northern part of the city, has witnessed a greater decrease in water supply, climate regulation, environmental purification, hydrological adjustment, soil conservation, biodiversity, and landscape than any other districts, demonstrating that the loss of its ESV was the most serious. At the same time, the ESVs of hydrological adjustment and environmental purification have increased in Qingpu District to the west of the city and its surrounding areas.

To a large extent, the change in land-use type from 2000 to 2020 in Shanghai has led to a decrease in the overall urban ecosystem service supply, especially regarding the reduction in the value of biodiversity (−407.65 million yuan). In fact, similar situations have been witnessed worldwide [87,88,89]. Among the 11 ecosystem services in this study, the value of the ecosystem services demonstrated a downward trend to varying degrees, barring the value of water supply and hydrological adjusting, has partially improved. This trend is often accompanied by the expansion of urban construction land, and is consistent with the results of previous studies. That is, the continuous expansion of cities has a negative impact on the provision of other ecosystem services such as cycling, climate regulation, genetic resources, soil fertility, and water regulation [54]. At the same time, from the perspective of differences in ecosystem structure and function, although the increase in construction land expands the spatial scope of terrestrial ecosystems, it also damages the functions of other ecosystems [70]. Specifically, it affects the performance of urban ecosystem functions in many areas, such as climate regulation, water resource conservation, and biodiversity maintenance, such that it has a negative impact on the total ecosystem services value in Shanghai.

In fact, the policy is also an important factor that affects the change of urban LULC and leads to the difference of ESV. For example, in the Shanghai Main Functional Area Planning issued by the Shanghai Government, the development strategy for the vast area in the west and south of the city is divided into new areas, whose development focus is intensive and efficient, with improved functions, a friendly environment, a harmonious society, and the integration of urban and rural areas. In other words, the increase in lakes in Shanghai is probably due to the promotion of relevant policies, this change of LULC in turn led to the improvement of ESV in Qingpu District. Overall, while working to improve urban economic development and adjust the structure of urban land use, decision-makers and administrators should recognize the relationship between different land use types and the ecosystem services provided by them, which contribute to the rational layout of cities, sustainable use of land, and management of natural ecosystems.

### 4.2. Limitations of the Study and Area of Future Research

Based on the land use classification (GB/T 21010-2017) and relevant calculation rules of ESV and combined with the characteristics of land types in Shanghai, this study focused on the land use types that can highlight the characteristics of the study region, such as cultivated land, forestland, grassland, trench and lakes, wetland pits, and construction land. Construction land with ESV equivalent to less than 0 was excluded from this study. However, considering the expanding area for construction land in Shanghai year by year in recent years, it can be known that the actual loss of ESV in Shanghai may be higher than the research results, which is comparable to previous research results [80]. Meanwhile, socioeconomic factors, such as the willingness to convert land and change in land use policies during this period, also significantly impacts the calculation results of ESV. In addition, considering the uncertainty of the ecosystem and the biological community it represents, the existing sensitivity analysis methods regarding the reliability test of the estimated results can be improved further [90,91]. However, existing studies have not reached a consensus on the robustness test of ESV, and the limitations of this study also need to be considered and jointly solved by other relevant studies.

According to the rule that the value of an equivalent factor is equal to 1/7 of the market value of grain output per unit area in the same year, the value change caused by different prices in different periods of time is eliminated by using the CPI index to revise the ESV coefficient of land use types in Shanghai, which makes the calculated results closer to the actual ESV and improves the accuracy and comparability of value evaluation, thus properly preserving and developing the traditional methods. In general, this study selected Shanghai as a representative city to consider land use/land cover and its influence mechanism on ecosystem services value, and used TM remote sensing image data and relevant statistical data to objectively indicate the impact of LULC on ESV in Shanghai from 2000 to 2020 through indicators such as dynamic degree of land use, ESV and its contribution rate, and CS. It provides theoretical and methodological support for promoting the sustainable development of land resources and the social economy in Shanghai by enhancing a deeper and more comprehensive understanding of issues related to ESV in rapidly urbanized areas, thus providing an important reference for decision-making regarding the rational layout of cities, sustainable use of land, and management of natural ecosystems.

## 5. Conclusions

Since its reform and ’opening up’, China has made remarkable achievements in economic and social development. With rapid economic growth and urban development, capital, labor force, land, and other traditional elements continue to gather in cities for urban development in all aspects, rendering an increasingly limited use of urban land and thus enhancing the impact of land use change on the ecosystem. Based on existing research results and the characteristics of LULC and ESV in Shanghai, this study comprehensively considered the actual situation of the city, calculated the equivalent factor of ESV in Shanghai, and then measured the ESV and its changes in Shanghai from 2000 to 2020.

As a representative of China’s rapid urbanization, Shanghai has undergone significant changes in land use structure during the process of urban expansion, which is mainly reflected in the encroachment of the expanding construction land on cultivated land, forest land, trench, and lakes, as well as other land types. The LULC pattern in Shanghai rapidly changed from 2000 to 2020, in particular, the area of cultivated land has shrunk by 123.96 thousand hm^2^, while the construction land has expanded by 141.26 thousand hm^2^, which led to a decline in ESV of the entire city, especially regarding hydrological adjustment and biodiversity, which were reflected in the declining trend of the value generated by various ecosystem services. In particular, the contribution of wetland pits to the overall ecosystem of Shanghai cannot be ignored, and trench and lakes are essential for improving ecosystem services: although the area of trench and lakes only occupies 1.67–3.16% of the total area of land, its ecological value accounts for an astonishing 23.80–50.70% of the total ESV. As such, they cannot be ignored despite the small fraction they occupy in the entire area. In the future planning of land use in Shanghai, we should heed to the ecological service function of land types, such as trench, lakes, and wetlands pits, and clarify the key role they play in maintaining the balance and healthy development of urban ecosystems. At the district level, barring Chongming District, the value of water supply has increased in all other districts, especially Qingpu District (+118.65 million yuan) in the west of the city. Meanwhile, the main reason for the decrease of ecosystem services value from 2000 to 2020 was the decrease of ecosystem services value in Chongming District in the north and Pudong New Area in the east, which experienced the most severe loss of ESV. However, by virtue of the overall planning of the city and the advantage of its resource endowment, the value of hydrological adjustment in Qingpu District has risen significantly (+1011.45 million yuan) and that of environmental purification has also increased (+30.87 million yuan). We believe that the scale of urbanization and industrialization should be set and controlled in terms of time and space to prevent the pollution and destruction of wetland pits, trench, and lakes during urban development. However, the results of the sensitivity analysis demonstrate that ESV is inelastic to the ecosystem services value coefficient (V), which is consistent with the realities of Shanghai, indicating the reliability of the ESV coefficient determined in this study.

## Figures and Tables

**Table 1:** Description of LULC Types in Shanghai.

LULC Types		Descriptions
Cultivated land		Refer to the land for planting crops, including arable land, newly reclaimed land, recreational land, rotation and rest land, grass field rotation crop land; Land for fruit, crop, agriculture, and forestry; Beaches and tidelands cultivated for more than three years.
Forestland		Refer to the land for growing trees, shrubs, bamboo, as well as coastal mangrove forest and other forestry land.
Grassland		Refer to all kinds of grassland, mainly herbaceous plants, with the coverage above 5%, including shrub grassland for grazing and sparsely forested grassland with canopy density below.
Trench and lake ^1^	Trench	Refer to the natural or artificial excavation of rivers and area with the bulk perennially below water level. Artificial channels include embankments.
	Lakes	Refer to the land in naturally waterlogged area that is permanently below water level.
Wetland pits ^2^	Swag	Refer to the land in artificial water storage area that is perennially below the water level.
	Ocean beaches	Refer to the tidal zone between high tide and low tide of coastal spring tide.
	Bottomland	Refer to the land between the water level of a river or lake and the water level of a flood.
Construction land		Refer to urban areas, residential areas and land for industrial, mining and transportation purposes.

^1^ Trench and lakes include rivers, ditches, and lakes. ^2^ Wetland pits include ponds, beaches, and beach lands.

**Table 2:** Single equivalent ecological service value in the Shanghai Ecosystem.

Year	2000	2005	2010	2015	2020
Output value of food crops agricultural commodities (×108 yuan)	10.15	9.20	16.43	21.69	24.04
Sown area of grain Crops (×104 hm^2^)	25.88	16.61	20.12	18.13	11.43
Economic value of grain crops (yuan hm^−2^)	3921.95	5538.83	8166.00	11,963.60	21,032.37
Single equivalent ecosystem service value (yuan hm^−2^)	560.28	820.63	1372.16	2338.35	4571.75

**Table 3:** Ecosystem service value coefficient of land use types in Shanghai (yuan hm^−2^).

Ecosystem Service Types	Cultivated Land	Forest Land	Grassland	Trench and Lakes	Wetland Pits
Supply service					
Food production	2135.56	487.99	734.40	985.64	1546.10
Material production	473.49	1120.93	1082.27	966.32	444.50
Water supply	−2522.08	579.79	599.12	5005.51	16,021.50
Regulating service					
Gas regulation	1720.04	3686.49	3807.28	3672.00	1488.13
Climate regulation	898.67	11,030.49	10,069.00	6957.47	4425.72
Environment purification	260.91	3232.32	3324.12	6957.47	10,726.10
Hydrological adjusting	2889.28	7218.37	7382.65	46,827.62	197,592.09
Support service					
Soil conservation	1004.97	4488.53	4638.31	4464.38	1797.35
Nutrient cycling	299.56	343.04	347.87	347.87	135.28
Biodiversity	328.55	4087.51	4213.13	15,209.80	4928.21
Cultural service					
Landscape	144.95	1792.51	1855.32	9141.34	3652.67

**Table 4:** Change of land use types in Shanghai from 2000 to 2020.

Index	Year	Cultivated Land	Forest Land	Grassland	Trench and Lakes		Wetland Pits			Construction Land
					Trench	Lakes	Swag	Ocean Beaches	Bottomland	
Area (Thousand hm^2^)	2000	455.21	10.31	1.17	10.98	0.26	31.04	7.52	9.37	144.98
	2005	423.89	11.13	1.89	10.96	0.25	27.10	5.06	5.79	184.77
	2010	377.47	10.15	1.88	10.50	5.97	21.12	4.87	5.86	233.19
	2015	359.98	9.84	1.35	9.26	5.51	21.78	15.32	6.55	254.94
	2020	331.25	10.93	6.74	15.20	6.36	16.14	0.14	2.48	286.23
	2000–2020	−123.96	0.62	5.57	4.22	6.10	−14.90	−7.38	−6.89	141.26
Area proportion (%)	2000	67.86	1.54	0.17	1.64	0.04	4.63	1.12	1.40	21.61
	2005	63.19	1.66	0.28	1.63	0.04	4.04	0.75	0.86	27.54
	2010	56.25	1.51	0.28	1.57	0.89	3.15	0.73	0.87	34.75
	2015	52.59	1.44	0.20	1.35	0.80	3.18	2.24	0.96	37.24
	2020	49.04	1.62	1.00	2.25	0.94	2.39	0.02	0.37	42.38
*LUDD_i_*	2000–2005	−0.01	0.02	0.12	0.00	0.00	−0.03	−0.07	−0.08	0.05
	2005–2010	−0.02	−0.02	0.00	−0.01	4.50	−0.04	−0.01	0.00	0.05
	2010–2015	−0.01	−0.01	−0.06	−0.02	−0.02	0.01	0.43	0.02	0.02
	2015–2020	−0.02	0.02	0.80	0.13	0.03	−0.05	−0.20	−0.12	0.02
	2000–2020	−0.01	0.01	0.24	0.02	1.20	−0.02	−0.05	−0.04	0.05

**Table 5:** Estimated values of ESV in Shanghai from 2000 to 2020.

Index	Year	Cultivated Land	Forest Land	Grassland	Trench and Lakes		Wetland Pits			Total
					Trench	Lakes	Swag	Ocean Beaches	Bottomland	
ESV (Million yuan)	2000	3475.02	392.44	44.56	2665.13	61.92	3120.79	756.46	942.44	11,458.78
	2005	3235.95	423.52	71.86	2661.37	61.63	2724.55	509.03	581.81	10,269.72
	2010	2881.58	386.44	71.43	2549.75	1448.38	2123.18	489.85	588.97	10,539.58
	2015	2748.04	374.62	51.42	2247.48	1337.72	2189.89	1540.25	658.98	11,148.40
	2020	2528.73	416.16	256.38	3689.42	1543.42	1622.67	14.17	249.42	10,320.38
	2000–2020	−946.29	23.72	211.81	1024.29	1481.50	−1498.12	−742.30	−693.02	−1138.40
CRj (%)	2000	30.33	3.42	0.39	23.26	0.54	27.23	6.60	8.22	-
	2005	31.51	4.12	0.70	25.91	0.60	26.53	4.96	5.67	-
	2010	27.34	3.67	0.68	24.19	13.74	20.14	4.65	5.59	-
	2015	24.65	3.36	0.46	20.16	12.00	19.64	13.82	5.91	-
	2020	24.50	4.03	2.48	35.75	14.96	15.72	0.14	2.42	-
	2000–2020	−5.82	0.61	2.10	12.49	14.41	−11.51	−6.46	−5.81	-

**Table 6:** ESV changes caused by LULC in Shanghai from 2000 to 2020 (million yuan).

**(a) 2000–2005**								**(b) 2005–2010**							
**Year**		**2005**						**Year**		**2010**					
		**(1)**	**(2)**	**(3)**	**(4)**	**(5)**	(6) *			**(1)**	**(2)**	**(3)**	**(4)**	**(5)**	**(6)**
2000	(1)	0.00	34.98	0.21	12.10	175.26	−298.22	2005	(1)	0.00	15.21	1.16	88.67	122.10	−392.85
	(2)	−4.59	0.00	0.00	4.73	2.04	−18.70		(2)	−13.20	0.00	0.00	6.65	3.08	−46.02
	(3)	−2.03	0.00	0.00	0.00	1.09	−0.27		(3)	−1.74	0.00	0.00	2.10	1.73	−0.46
	(4)	−18.94	−0.39	−0.02	0.00	−2.10	−10.75		(4)	−70.97	−7.28	−1.40	0.00	−68.88	−113.68
	(5)	−750.19	−6.03	−62.17	3.64	0.00	−278.87		(5)	−116.62	−4.72	−2.42	841.97	0.00	−85.19
	(6)	18.83	2.70	0.04	6.01	2.33	0.00		(6)	39.54	5.31	0.17	50.82	10.41	0.00
**(c) 2010–2015**								**(d) 2015–2020**							
**Year**		**2015**						**Year**		**2020**					
		**(1)**	**(2)**	**(3)**	**(4)**	**(5)**	**(6)**			**(1)**	**(2)**	**(3)**	**(4)**	**(5)**	**(6)**
2010	(1)	0.00	0.00	0.00	47.76	31.19	−145.93	2015	(1)	0.00	15.36	86.66	380.41	221.34	−295.74
	(2)	0.00	0.00	0.00	0.00	0.00	−11.82		(2)	−17.64	0.00	−0.01	17.78	8.20	−28.78
	(3)	−11.40	0.00	0.00	0.00	1.59	−4.78		(3)	−3.30	0.00	0.00	4.96	3.72	−2.37
	(4)	−79.67	0.00	0.00	0.00	−221.07	−4.15		(4)	−104.39	−8.23	−8.25	0.00	−95.56	−113.61
	(5)	−109.29	0.00	0.00	0.00	0.00	−6.42		(5)	−1204.05	−3.28	−245.34	90.85	0.00	−307.33
	(6)	2.08	0.00	0.00	1.51	1.57	0.00		(6)	71.93	5.00	46.11	152.65	47.46	0.00

* (1)–(6) refer to cultivated land, forestland, grassland, trench and lakes, wetland pits, and construction land, respectively.

**Table 7:** Composition of ecosystem service value in Shanghai from 2000 to 2020.

Type of Service	Services Available	ESV (Million Yuan)					CR_i_ (%)				
		2000	2005	2010	2015	2020	2000	2005	2010	2015	2020
Supply service	Food production	1042.64	966.81	869.30	840.41	769.51	9.10	9.41	8.25	7.54	7.46
	Material production	279.68	256.88	230.24	231.69	204.10	2.44	2.50	2.18	2.08	1.98
	Water supply	−721.45	−691.83	−521.71	−446.24	−385.80	−6.30	−6.74	−4.95	−4.00	−3.74
Regulating service	Gas regulation	1018.20	933.36	835.30	842.89	736.69	8.89	9.09	7.93	7.56	7.14
	Climate regulation	917.85	836.36	764.58	814.77	712.05	8.01	8.14	7.25	7.31	6.90
	Environment purification	610.02	537.19	535.78	592.38	505.90	5.32	5.23	5.08	5.31	4.90
	Hydrological adjusting	5862.90	5312.52	5923.46	6083.63	6223.58	51.17	51.73	56.20	54.57	60.30
Support service	Soil conservation	743.39	674.28	605.41	633.65	535.72	6.49	6.57	5.74	5.68	5.19
	Nutrient cycling	158.50	146.17	130.52	128.87	114.77	1.38	1.42	1.24	1.16	1.11
	Biodiversity	981.15	825.20	739.01	900.99	573.50	8.56	8.04	7.01	8.08	5.56
Cultural service	Landscape	565.90	472.78	427.70	525.36	330.36	4.94	4.60	4.06	4.71	3.20

**Table 8:** Coefficient of sensitivity of ecosystem service value coefficient in Shanghai.

Value Coefficient	ESV (million yuan)					CS				
	2000	2005	2010	2015	2020	2000	2005	2010	2015	2020
Cultivated land (+50.0%)	13,196.29	11,887.70	11,980.37	12,522.41	11,584.74	0.303	0.315	0.273	0.246	0.245
Cultivated land (−50.0%)	9721.27	8651.75	9098.79	9774.38	9056.01					
Forestland (+50.0%)	11,655.00	10,481.48	10,732.80	11,335.71	10,528.46	0.034	0.041	0.037	0.034	0.040
Forestland (−50.0%)	11,262.55	10,057.96	10,346.36	10,961.08	10,112.30					
Grassland (+50.0%)	11,481.06	10,305.65	10,575.29	11,174.11	10,448.57	0.004	0.007	0.007	0.005	0.025
Grassland (−50.0%)	11,436.49	10,233.79	10,503.86	11,122.69	10,192.19					
Wetland pits (+50.0%)	13,868.63	12,177.42	12,140.58	13,342.95	11,263.51	0.421	0.372	0.304	0.394	0.183
Wetland pits (−50.0%)	9048.93	8362.03	8938.58	8953.84	9377.24					
Trench and lakes (+50.0%)	12,822.30	11,631.23	12,538.64	12,941.00	12,936.80	0.238	0.265	0.379	0.322	0.507
Trench and lakes (−50.0%)	10,095.25	8908.22	8540.51	9355.79	7703.96					

## Data Availability

In this study, the data used to analyze the impact of LULC on ESV in Shanghai are based on five land-use maps generated by the Institute of Geographic Sciences and Natural Resources Research, CAS (https://www.resdc.cn/ (accessed on 20 July 2022)).
